# How we use the neurological pupil index (NPi)

**DOI:** 10.1186/s13054-026-06177-5

**Published:** 2026-07-03

**Authors:** Giuseppe Citerio, Fabio Silvio Taccone

**Affiliations:** 1 School of Medicine and Surgery, University of Milano-Bicocca, Milano, Italy; 2 NeuroIntensive Care Unit, Neuroscience Department, Fondazione IRCCS San Gerardo dei Tintori, Monza, Italy; 3 Department of Intensive Care, Hôpital Universitaire de Bruxelles, Université Libre de Bruxelles, Brussels, Belgium

In neurocritical care, pupillary abnormalities remain among the few clinical signs that remain reliable when consciousness, motor responses, sedation, mechanical ventilation or neuromuscular blockade make the rest of the neurological examination difficult to interpret. The estimation of pupillary diameter and of the pupillary light reflex (PLR) is simple, fast and immediately available at the bedside [1]. However, the clinical interpretation of pupillary abnormalities remains challenging, as impaired or asymmetric pupillary responses may arise from a wide range of neurological and non-neurological conditions. From a central nervous system perspective, abnormal pupillary reactivity or asymmetry may reflect trans-tentorial herniation, compression or dysfunction of the oculomotor nerve, midbrain injury, posterior fossa pathology, or diffuse hypoxic–ischemic damage affecting brainstem structures. However, pupillary abnormalities are not specific to intracranial pathology; peripheral causes, including ocular trauma, optic nerve injury, pre-existing ophthalmological disease, previous ocular surgery, and exposure to mydriatic or miotic medications, or even seizures, may result into specific pupillary abnormalities. Consequently, pupillary assessment should always be interpreted within the broader clinical context and integrated with other neurological, imaging and physiological data [1]. As such, new anisocoria or loss of reactivity in a deeply sedated patient with acute brain injury should be treated as a warning sign until proven otherwise; anisocoria in an awake patient may instead reflect a chronic or ocular condition and requires a different diagnostic frame.

In this setting, manual penlight examination remains useful, but it has obvious limitations [2]. Small variations in pupillary dynamics, such as latency, constriction amplitude, constriction velocity, dilation velocity, and limited anisocoria, are challenging to quantify accurately by visual inspection alone. As a result, their detection is subject to considerable interobserver variability and limited reproducibility during longitudinal neurological assessment [3]. Automated pupillometry was developed to transform this traditional neurological assessment into an objective, reproducible, and quantifiable parameter that can be reliably followed over time at the bedside [4–6]. In contrast to subjective visual assessment, automated devices provide precise measurements of multiple aspects of the PLR, including baseline pupil diameter, minimum pupil size after light stimulation, constriction amplitude (or percentage constriction), latency to constriction, average and maximum constriction velocity, dilation velocity, and recovery characteristics. In addition to reporting individual pupillometric variables, some devices generate integrated proprietary metrics, such as the Neurological Pupil index (NPi). The NPi combines several dynamic features of the PLR, including baseline pupil size, latency, constriction amplitude, constriction velocity, and dilation velocity, into a normalized measure of pupillary reactivity [4, 5]. These parameters are compared against a manufacturer-derived normative database and subsequently transformed into a standardized score [6]. Accordingly, the NPi should be interpreted as a validated composite indicator of the functional integrity of the afferent and efferent pupillary pathways rather than as a direct measurement of pupil size or a single physiological pupillometric variable.

The practical question is not whether NPi is useful, but how to implement it in clinical practice. In our setting, NPi is most useful when the neurological examination matters but is incomplete or difficult to reproduce: unconscious patients, deeply sedated or mechanically ventilated patients, patients receiving neuromuscular blockade, patients with acute brain injury at risk of neurological deterioration [6–8]. A particularly important application is also post-anoxic coma after cardiac arrest, where quantitative pupillometry and NPi significantly contribute to multimodal prognostication [7]. In these patients, we commonly repeat measurements every 4–6 h, and more frequently when there is clinical concern or after a therapeutic intervention. Bedside nurses can perform the measurements reliably after a short learning curve, provided that eye opening, device position, acquisition quality and waveform morphology are checked systematically.

At the bedside, the NPi can then be viewed as a quantitative measure of the extent to which an individual PLR differs from the expected physiological response. When a detectable pupillary reaction to light is present, the device generates an NPi value on a proprietary scale ranging from 0.1 to 4.9 [5, 6]. In clinical practice, values ≥ 3 are generally considered indicative of normal pupillary reactivity, whereas values between 0.1 and 2.9 suggest an abnormal response. Although the exact algorithm remains proprietary, the NPi is derived by comparing multiple dynamic features of the pupillary light reflex against a normative reference database. Conceptually, an NPi value of approximately 3 represents the lower limit of the expected physiological range, corresponding to a response that deviates substantially from the normative mean, roughly equivalent to three standard deviations below normal values. As the NPi decreases further, the degree of pupillary dysfunction becomes progressively more pronounced, reflecting increasing impairment of the neural pathways involved in the PLR [5–7]. NPi should therefore not be interpreted as a refined measure of pupil size. Diameter is only one component of the response. A small pupil may be reactive, and a large pupil may still constrict normally. Opioids may reduce pupil size without implying brainstem dysfunction. The clinical value of NPi lies in the integration of dynamic features of the light reflex, not in diameter alone [5]. The NPi value of zero deserves special attention, as it is not simply the lowest value on the same continuous scale but indicates the absence of a measurable light response. In practical terms, this is a categorical finding; a pupil with NPi 1.8 is abnormal, as are all values below 3. A pupil with NPi = 0 is non-responsive according to the device measurement and should be treated as a distinct and more severe finding. When we see a NPi of 0, we immediately repeat the measurement, inspect the waveform, check eye opening and device alignment, review the ocular history, compare the contralateral eye, and interpret the finding in the broader neurological and systemic context [3].

This is also how we interpret isolated versus repeated abnormalities. A single abnormal value should not be ignored, but it should first trigger reassessment. The prognostic and clinical meaning becomes stronger when the abnormality is persistent, progressive, unilateral, newly asymmetric, or associated with other signs of neurological deterioration. This issue was addressed by the ORANGE study, a prospective, observational, multicentre international cohort study evaluating whether serial NPi measurements during the first week after severe non-anoxic acute brain injury were associated with 6-month neurological outcome and mortality. The ORANGE results do not imply that an isolated abnormal NPi value is clinically irrelevant; rather, they show that prognostic information becomes stronger when abnormality is considered as a repeated and cumulative exposure over time [6]. This supports the way many clinicians already use pupillometry at the bedside: not as a single number, but as a trajectory.

Individual pupillary variables are influenced by sedatives, analgesics, opioids, ambient conditions and technical factors. Baseline diameter, constriction amplitude, latency and velocities may all change for reasons that are not directly related to brainstem injury [1, 5, 9]. This makes isolated raw variables difficult to interpret at the bedside. The composite structure of NPi partly addresses this problem by integrating several components of the light reflex into a single normalized score and being less vulnerable to these effects [10, 11]. This is particularly relevant in mechanically ventilated, sedated or occasionally paralyzed patients, where conventional neurological assessment is pharmacologically limited. This does not mean that NPi is immune to drugs, physiology or technical problems. It means that, compared with the individual variables it integrates, it provides a more stable and interpretable signal.

Ambient light is another practical issue. Lighting conditions may influence quantitative pupillometry, and standardized measurement conditions remain preferable when feasible. However, the magnitude of these effects is usually small [12, 13]. Minor variations, such as a change from 4.6 to 4.3, should rarely drive clinical decisions in isolation. A progressive fall, such as from 4.4 to 3.2 and then to 2.4, especially if unilateral or sustained, is different. In that situation, the pattern rather than the absolute value should prompt concern.

Only after addressing these practical considerations can an important misconception be clarified: the NPi should not be interpreted as a surrogate measure of intracranial pressure (ICP). Although pupillary dysfunction may occur in the setting of intracranial hypertension, the relationship between NPi and ICP is neither direct nor consistent. The NPi primarily reflects the integrity of the afferent and efferent pathways involved in the pupillary light reflex, whereas ICP represents a complex physiological variable influenced by intracranial volume, compliance, and cerebral hemodynamics. Accordingly, abnormalities in one parameter do not necessarily imply abnormalities in the other. This distinction is supported by data from the ORANGE study, in which a secondary longitudinal analysis demonstrated that temporal changes in NPi and invasive ICP measurements were only weakly associated and did not evolve in parallel over time. These findings suggest that pupillary reactivity and intracranial pressure provide complementary, rather than interchangeable, information regarding neurological function and intracranial physiology [14]. As such, intracranial hypertension may occur without relevant brainstem or third-nerve pathway involvement, and NPi may remain normal. Conversely, NPi may be abnormal without elevated ICP, for example in optic nerve injury, ocular pathology, posterior fossa lesions, pharmacological effects or technical artefacts [1]. For this reason, isolated NPi measurements should not be used as a direct trigger for ICP-directed treatment.

A low or falling NPi should instead prompt a structured reassessment. First, repeat the measurement and confirm adequate eye opening, device alignment and waveform quality. Second, compare ipsilateral and contralateral pupil size, NPi values and waveform morphology. Third, review ocular history, local eye pathology, ocular trauma, previous ocular surgery, recent ophthalmic drugs, sedation, analgesia and systemic physiological conditions. Fourth, integrate the finding with neurological examination, oxygenation, ventilation, temperature, haemodynamics, ICP and cerebral perfusion pressure when available, and neuroimaging. If the abnormality is persistent, worsening, asymmetric or clinically concordant, escalation to urgent imaging or intervention should be considered.

Several additional caveats are important. NPi should not be considered synonymous with automated pupillometry in general. Other devices and other quantitative pupillometric variables may provide clinically relevant information. We use NPi not because it is necessarily superior to every other pupillometric approach, but because it is standardized, widely validated in neurocritical care, supported by multicenter clinical data, and easy to interpret serially on a normalized scale [6, 8]. Whether alternative indices or raw pupillometric variables provide equivalent or superior clinical information remains an important question for comparative research. The proprietary nature of the index should also be acknowledged. The final transformation into the NPi score is manufacturer-derived and cannot be independently reproduced from raw recordings using open-source code [4, 5]. For this reason, NPi should be regarded as a clinically validated composite index whose strengths are standardization, reproducibility and serial bedside use, rather than full algorithmic transparency.

Several limitations should also be acknowledged. Although abnormal NPi values and abnormal NPi burden are associated with neurological outcome in observational studies [6], there is currently no randomized evidence that NPi-guided management improves patient outcome. In studies where NPi is collected as part of routine care, treating clinicians may not always be blinded to the measurements. As with other neurological signs used for prognostication, this may leave some risk of self-fulfilling prophecy, particularly when decisions about limitation or withdrawal of life-sustaining treatment are considered. Formal medico-economic studies demonstrating that investment in automated NPi monitoring is offset by improved long-term outcomes or reduced healthcare costs are also lacking. These limitations do not negate the clinical value of quantitative pupillometry as a standardized bedside assessment, but they should temper claims about prognosis modification, resource allocation and device-specific superiority.

When used appropriately, the NPi does not reduce neurocritical care to a single numerical value. Rather, it provides an objective and reproducible quantification of a traditional neurological sign, allowing clinicians to track pupillary function consistently over time. In clinical practice, the greatest value of the NPi lies not in an isolated measurement but in its longitudinal behavior. Trends over time, the development of inter-eye asymmetry, the persistence of abnormalities, and their response to therapeutic interventions often provide more meaningful information than a single value obtained at one time point. Importantly, a low or declining NPi should not automatically trigger therapeutic intervention. Instead, it should prompt a systematic reassessment of the patient. Potential technical factors should first be excluded, followed by a physiological interpretation of the finding within the broader clinical context. Pupillary abnormalities must be integrated with the neurological examination, neuroimaging findings, systemic physiological variables, and, when available, invasive neuromonitoring data. In this way, the NPi serves as a complementary marker of neurological function that supports clinical decision-making rather than replacing clinical judgment (Fig. 1).

**Fig. 1:**
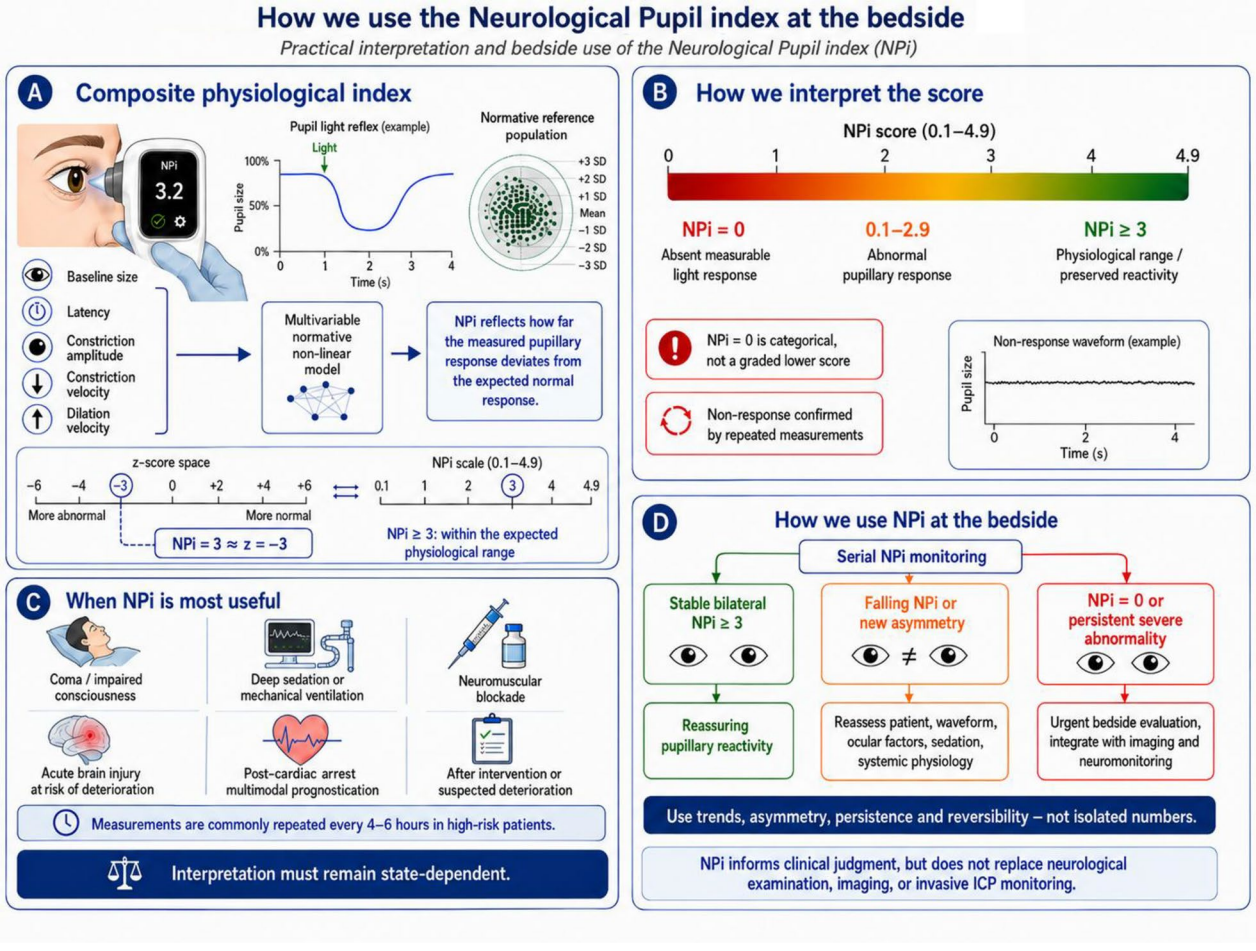
This schematic summarises the physiological basis, interpretation, and bedside use of the Neurological Pupil index (NPi) in patients with acute brain injury or impaired consciousness. **A** Composite physiological index. NPi is derived from automated pupillometry and integrates several components of the pupillary light reflex, including baseline pupil size, latency, constriction amplitude, constriction velocity, and dilation velocity. These measured variables are compared with a manufacturer-derived normative model and transformed into a standardised, device-specific score. NPi should therefore be interpreted as a validated composite index of pupillary pathway function rather than as a raw pupillometric variable, a simple measure of pupil size, or a fully transparent physiological measurement. **B** How we interpret the score. When a measurable light response is detected, NPi is reported on a device-specific scale from 0.1 to 4.9. Values ≥ 3 indicate preserved pupillary reactivity within the expected physiological range, whereas values from 0.1 to 2.9 indicate an abnormal pupillary response. By contrast, NPi = 0 indicates absence of a measurable light response. This should be treated as a categorical finding, not as the lowest point of the same continuous scale, and should be confirmed by repeat measurement and inspection of waveform quality. **C** When NPi is most useful. Serial NPi monitoring is particularly useful when the neurological examination is important but limited or difficult to reproduce: coma or impaired consciousness, deep sedation, mechanical ventilation, neuromuscular blockade, acute brain injury at risk of deterioration, post-cardiac arrest coma requiring multimodal prognostication, and reassessment after therapeutic interventions or suspected deterioration. In high-risk patients, measurements are commonly repeated every 4–6 h, and more frequently when there is clinical concern. Interpretation must remain state-dependent: new anisocoria or loss of reactivity in a comatose or sedated acute brain injury patient has a different meaning from chronic anisocoria in an awake patient. **D** How we use NPi at the bedside. NPi is best used as a serial monitoring variable rather than as an isolated number. Stable bilateral values ≥ 3 are generally reassuring. Falling values, new inter-eye asymmetry, repeated values < 3, or persistent NPi = 0 should prompt structured reassessment: repeat the measurement, inspect waveform quality, compare both eyes, check ocular and pharmacological confounders, review systemic physiology, and integrate the finding with neurological examination, neuroimaging, and invasive neuromonitoring when available. The key clinical information lies in trends, asymmetry, persistence, and reversibility. NPi informs clinical judgement but does not replace neurological examination, imaging, or invasive intracranial pressure monitoring.

## Data Availability

No datasets were generated or analysed during the current study.

## Declarations

### Use of artificial intelligence tools

Artificial intelligence–assisted tools, including ChatGPT, were used to support language editing, text refinement, and graphical development. These tools were not used to generate scientific concepts, data analysis, data interpretation, references, or conclusions. All scientific content, wording, figure content, and final interpretations were critically reviewed, edited, and approved by the authors, who take full responsibility for the accuracy and integrity of the manuscript.
